# XynDZ5: A New Thermostable GH10 Xylanase

**DOI:** 10.3389/fmicb.2020.00545

**Published:** 2020-04-24

**Authors:** Dimitra Zarafeta, Anastasia P. Galanopoulou, Maria Evangelia Leni, Stavroula I. Kaili, Magda S. Chegkazi, Evangelia D. Chrysina, Fragiskos N. Kolisis, Dimitris G. Hatzinikolaou, Georgios Skretas

**Affiliations:** ^1^Institute of Chemical Biology, National Hellenic Research Foundation, Athens, Greece; ^2^Department of Biology, Enzyme and Microbial Biotechnology Unit, National and Kapodistrian University of Athens, Athens, Greece; ^3^Laboratory of Biotechnology, School of Chemical Engineering, National Technical University of Athens, Athens, Greece

**Keywords:** xylanase, thermostability, mode of action, genome analysis, biocatalysis, biotechnology

## Abstract

Xylanolytic enzymes have a broad range of applications in industrial biotechnology as biocatalytic components of various processes and products, such as food additives, bakery products, coffee extraction, agricultural silage and functional foods. An increasing market demand has driven the growing interest for the discovery of xylanases with specific industrially relevant characteristics, such as stability at elevated temperatures and in the presence of other denaturing factors, which will facilitate their incorporation into industrial processes. In this work, we report the discovery and biochemical characterization of a new thermostable GH10 xylanase, termed XynDZ5, exhibiting only 26% amino acid sequence identity to the closest characterized xylanolytic enzyme. This new enzyme was discovered in an Icelandic hot spring enrichment culture of a *Thermoanaerobacterium* species using a recently developed bioinformatic analysis platform. XynDZ5 was produced recombinantly in *Escherichia coli*, purified and characterized biochemically. This analysis revealed that it acts as an endo-1,4-β-xylanase that performs optimally at 65–75°C and pH 7.5. The enzyme is capable of retaining high levels of catalytic efficiency after several hours of incubation at high temperatures, as well as in the presence of significant concentrations of a range of metal ions and denaturing agents. Interestingly, the XynDZ5 biochemical profile was found to be atypical, as it also exhibits significant exo-activity. Computational modeling of its three-dimensional structure predicted a (β/α)_8_ TIM barrel fold, which is very frequently encountered among family GH10 enzymes. This modeled structure has provided clues about structural features that may explain aspects of its catalytic performance. Our results suggest that XynDZ5 represents a promising new candidate biocatalyst appropriate for several high-temperature biotechnological applications in the pulp, paper, baking, animal-feed and biofuel industries.

## Introduction

Hemicelluloses comprise almost one third of all renewable organic matter on the planet and are the most abundant biopolymer group after cellulose (Saha, 2003; Soni et al., 2018). 20–40% of the hemicellulose content is xylan, an amorphous structural polysaccharide found in both hardwood and annual plants (Basit et al., 2018). Xylan consists of a xylose backbone, which is decorated with a source-dependent variety of side-chain substitutions. Owing to its high heterogeneity, complete degradation of xylan requires the synergistic action of different types of enzymes, which are cumulatively referred to as xylanases (Shallom and Shoham, 2003). In nature, xylanolytic enzymes are produced by organisms that are capable of utilizing pentoses, mainly xylose and arabinose, as carbon sources. Among the xylanolytic enzymes, endo-β-1,4-xylanases (EC 3.2.1.8) are the glycoside hydrolases (GH) that cleave the inner β-1,4 bonds between two xylose monomers in the xylan backbone and xylo-oligosaccharides (XOs) (Chakdar et al., 2016). According to amino acid sequence similarities, endo-β-1,4-xylanases are classified into different GH families, with most of them belonging to families GH10 and GH11 (Chakdar et al., 2016). The GH10 family includes mainly endo-1,4-β-xylanases originating from all three domains of life. They exhibit versatile substrate specificity, as they are often able to hydrolyse low-molecular-weight cellulosic substrates (Juturu and Wu, 2012). In terms of structure, GH10 xylanases typically fold into a (β/α)_8_ triosephosphate isomerase (TIM) barrel conformation (Collins et al., 2005). Contrary to GH10, most GH11 family enzymes are fungal and bacterial endo-β-1,4-xylanases (EC 3.2.1.8) that cleave internal β-1,4-xylosidic bonds, acting exclusively on D-xylose-containing substrates without hydrolytic activity against cellulose. GH11 xylanases typically fold into a β-jelly roll architecture (Paes et al., 2012).

X ylanolytic enzymes play a key role in biotechnology since they comprise a large part of the industrially exploited hydrolases, a category of enzymes corresponding to about 75% of the market share of all industrial biocatalysts (Acharya and Chaudhary, 2012). Besides their extensive use in the pulp and paper industries (Guerriero et al., 2015), more novel applications of xylanases include their use as food additives in poultry farming; enhancers of baked products; extracting agents of plant pigments, oils and coffee; brewing and processing agents of wine, beer and fruit juices; bioconversion catalysts of agricultural residues, and more (Chakdar et al., 2016). More recently, xylanases have also been utilized by the pharmaceutical industry for the production of xylooligosaccharides for use as prebiotics with established health-promoting properties (Aachary and Prapulla, 2011). So far, xylooligosaccharides are the only nutraceutical originating from agricultural residues, thus creating a large and rapidly growing market (Samanta et al., 2015). For many such industrial applications, high temperatures in the range from 60 to 90°C are often an integral part of the process (Sani and Krishnaraj, 2017). Heat-tolerant xylanases are required for optimal bioprocessing in the pulp and paper and biorefinery industries, where the raw materials are initially pre-treated at elevated temperatures. As a result, thermophilic enzyme use eliminates the need for cooling prior to the addition of the biocatalyst, thus saving significant amounts of time and energy (Kumar et al., 2016; Rakotoarivonina et al., 2016). Furthermore, higher operation temperatures, in general, enhance substrate and product solubility, minimize diffusion resistance, reduce pumping costs due to reduced viscosity, and minimize the risk of microbial contaminations. Apart from being part of the production pipeline, thermostable xylanases can also be utilized as a constituent of an end-product intended for use at high temperatures, such as bakery mixtures and detergents. Due to their increasing demand, the market share of these enzymes, which is now 200–300 million dollars, is estimated to increase further and reach 500 million dollars by 2023 (Chadha et al., 2019).

Following this demand for new and improved thermostable xylanases, which is well reflected in the growing number of patents applications for such enzymes (Soni and Kango, 2013), researchers have set the spotlight on thermophilic, hemicellulose-degrading bacteria and archaea as a natural source of potential thermostable xylanolytic biocatalysts (Basit et al., 2018). Such organisms are encountered in habitats with temperatures above 60°C, where they degrade plant biomass through the action of an extensive repertoire of enzymes, mainly GHs. Environmental sampling followed by culture-free or culture-dependent DNA extraction and functional *in vivo* screening or bioinformatic analysis, have proven powerful strategies toward the discovery of industrially relevant biocatalysts (Zarafeta et al., 2016b, c; Wohlgemuth et al., 2018).

In this study, we aimed to identify new thermostable xylanolytic enzymes with properties suited for industrial applications. Initially, we carried out a culture enrichment approach to select for xylan-degrading microorganisms, using an environmental sample collected from a hot spring located in Iceland. DNA isolated through this approach was sequenced and screened for genes encoding for putative xylanolytic enzymes. This procedure resulted in the discovery of XynDZ5, a new thermostable xylanase with very low sequence similarity to known xylanolytic enzymes. The new enzyme was cloned, overexpressed in *Escherichia coli*, and characterized biochemically. XynDZ5 exhibits biochemical characteristics that render it a promising biocatalyst for high-temperature biotechnological applications.

## Results

### Genome Analysis and Discovery of *xynDZ5*

**FIGURE 1:**
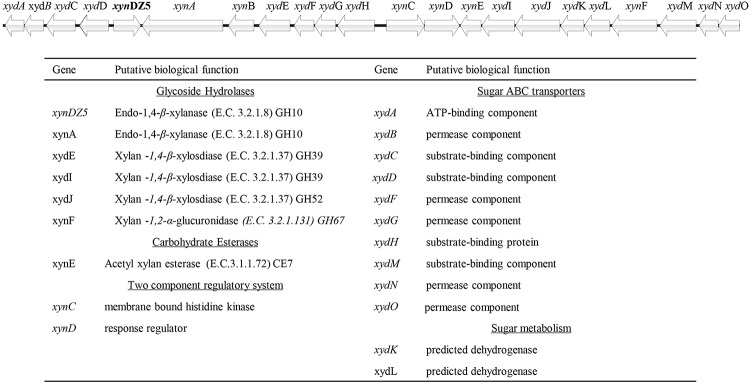
Schematic representation of the putative xylan degradation and utilization genetic locus of the *Thermoanaerobacterium* sp. found in the sequenced genomic material of the enrichment sample. Each arrow of the graph represents a gene in the locus **(upper part)**. The functional annotation of these genes is given at the table **(lower part)**.

In order to identify novel xylanolytic enzymes, we sampled the outflow of a hot spring in Grensdalur, Iceland and enriched it anaerobically in 0.5% (w/v) xylan. As previously described (Zarafeta et al., 2016a), a pure isolate was acquired and its genome was sequenced. Based on its 16S rRNA sequences, the strain belongs to the genus *Thermoanaerobacterium* (99% sequence identity). The sequencing reads were also assigned to the microbial taxa *Thermoanaerobacterium thermosaccharolyticum* or *Thermoanaerobacterium xylanolyticum*, thereby demonstrating that the isolate corresponds to a *Thermoanaerobacterium* species. Among the 2,822 putative protein-encoding genes obtained, 94 CAZy hits were detected, which corresponded to 53 distinct CAZy families: 29 GHs, ten glycosyl transferases (GTs), four carbohydrate esterases (CEs), one polysaccharide lyase (PL) and nine carbohydrate-binding modules (CBMs). As anticipated, many of the detected families were related to xylan degradation. In particular, members of the families of glycoside hydrolases GH3, GH5, GH10, GH26 and GH51 can putatively act as endo-1,4-β-xylanases (E.C. 3.2.1.8), members of the family GH26 can additionally act as endo-1,3-β- xylanases (E.C 3.2.1.32), while members of the families GH1, GH3, GH5, GH39, GH51, GH52 and GH120 contain putative 1,4-β*-*xylosidases (E.C. 3.2.137). Seven of the genes encoding for the above putative xylanolytic enzymes (*xynDZ5*, *xynA*, *xydE*, *xydI*, *xydJ*, and *xynF*) were encountered in the same 33.6-kb cluster, which contained additional putative genes responsible for the degradation and utilization of xylan and its derivatives (Figure 1). Besides the seven putative xylanolytic hydrolases described above, this 33.6 kb cluster also contained one putative two-component sensing system (*xynCD*); three ABC transporters, possibly related to the transfer of the occurring xylose and oligomers into the cell (*xydABCD*, *xydFGH*, *xydMNO*), and two oxidoreductases possibly related to the metabolism of the produced sugars (*xydK*, *xydL*).

**FIGURE 2:**
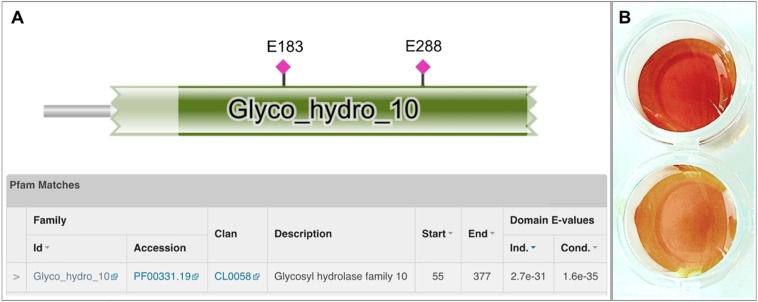
Discovery of the xylanolytic enzyme XynDZ5. **(A)** The 430-amino acid sequence of the putative xylanolytic enzyme corresponding to the *xynDZ5* ORF was analyzed against the Pfam-A database using HHMER. The analysis revealed that the predicted sequence contains a GH10 catalytic domain spanning amino acids 55–377 (green color). Also, the sequence was annotated to the TIM barrel GH superfamily (Clan CL0058), which contains a range of GHs that possess a TIM barrel fold. Two predicted catalytic residues were also detected in the XynDZ5 sequence at positions E183 and E288, as indicated. **(B)** Detection of xylanolytic activity by DNS assay and xylan as a substrate where the observed color change indicates the release of reducing sugars due to xylanolytic activity. *E. coli* cell lysates producing XynDZ5 from pASK75-*xyn*DZ5 gave a positive reaction (upper part), cell lysates of the same bacteria carrying an empty pASK75 vector were used as a negative control (bottom).

Among the detected genes encoding for putative xylanolytic enzymes, *xynA* and *xynDZ5* encode for putative hydrolases of the GH10 family, the main class of bacterial xylanases. *xynA* encodes for a multi-domain xylanase with more than 90% identity along the entire length of an endo-xylanase from *Thermoanaerobacterium saccharolyticum* [UniProtKB/Swiss-Prot: P36917] (Lee et al., 1993). On the other hand, XynDZ5 exhibits only 26% sequence identity and 73% query coverage to a previously characterized GH10 endo-xylanase from *Thermotoga neapolitana* [UniProtKB/Swiss-Prot: Q60041.1] (Velikodvorskaya et al., 1997) and, thus, was selected for further investigation. The sequence of this putative protein is 430 amino acids long with a predicted molecular mass of 49.9 kDa. XynDZ5 contains a GH10 domain and is not predicted to contain transmembrane regions or signal peptide sequences (Figure 2A). Based on its similarity to the endo-1,4-β-xylanase XynZ of *Clostridium thermocellum* [UniProtKB/Swiss-Prot: P10478] (Grepinet et al., 1988), XynDZ5 has two predicted catalytic residues at positions E183 and E288 (Figure 2A).

### Overexpression, Purification and Biochemical Characterization of XynDZ5

In order to study the biochemical properties of the new enzyme, XynDZ5 was recombinantly produced and purified. SDS-PAGE of the overexpressed enzyme in cell lysates and in isolated form indicated that its apparent molecular mass is ∼50 kDa (Supplementary Figure 1), which is in accordance with its calculated size. To test the xylanolytic activity of the overexpressed protein, cell extracts from *E. coli* cells carrying either pASK75-*xyn*DZ5 or empty vector as negative control were evaluated in a standard 3,5–dinitrosalicylic acid (DNS) xylanase activity assay (see Materials and Methods). Only the extracts from *xynDZ5-*overexpressing cells exhibited a color change (Figure 2B), thus indicating that XynDZ5 possesses xylanolytic activity.

**FIGURE 3:**
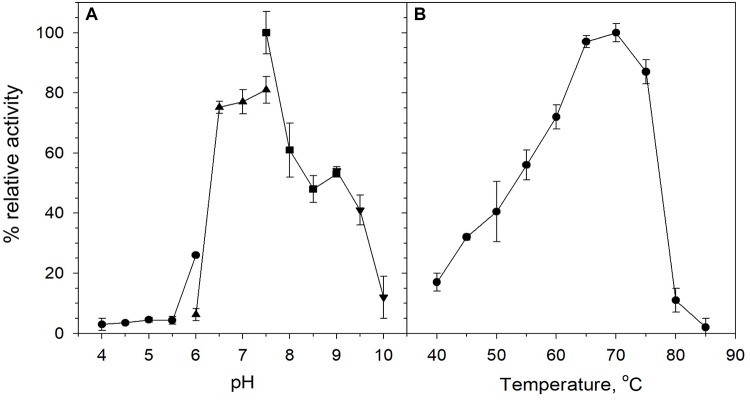
Effect of pH and temperature on the activity of XynDZ5. **(A)** Effect of pH on XynDZ5 activity. For the various pH values ranging from 4 to 10, the following buffer systems were used: acetate (∙), phosphate (▲), Tris–HCl (■), glycine-NaOH (▼). **(B)** Effect of temperature on XynDZ5 activity. Enzyme activity was measured at temperatures ranging from 40 to 85°C and pH 7.5 in the standard assay. The reported values correspond to the mean value from three independent experiments performed in triplicate ± one standard deviation from the mean value.

Biochemical characterization was carried out using purified protein and beechwood xylan as a model substrate. The activity of the enzyme was examined in the pH range 4–10. The enzyme retained high levels of catalytic activity at pH 6.5–9, with its optimal activity recorded at pH 7.5 (Figure 3A). At pH values below 6 and above 9, XynDZ5 activity was rapidly diminished, indicating the moderately alkalophilic profile of the enzyme. This is in accordance with the fact that bacterial thermostable xylanases, the majority of which belong to the GH10 family, act optimally at neutral to alkaline pH (Chakdar et al., 2016).

Measurements of the relative catalytic activity of the new enzyme at different temperatures revealed that XynDZ5 is a thermophilic enzyme that exhibits maximal activity at temperatures in the range 65–75°C (Figure 3B). At 60°C, the enzyme exhibited over 70% of its relative activity, while at 55, 50, and 45°C, the relative activity of XynDZ5 was reduced to 55, 40, and 30% of its maximal value, respectively. From temperatures 80°C and above, the enzyme showed practically no activity (Figure 3B).

**FIGURE 4:**
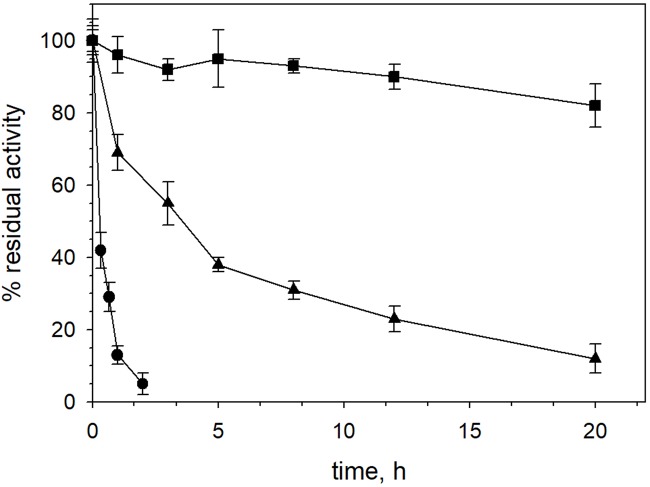
XynDZ5 thermostability. The thermostability of XynDZ5 was evaluated by measuring residual activity following incubation of the enzyme at 65°C (■), 70°C (▲), and 75°C (∙) for up to 20 h. The reported values correspond to the mean value from three independent experiments performed in triplicate ± SD.

XynDZ5 tolerated well prolonged exposure to elevated temperatures. At temperatures up to 65°C, the enzyme retained over 80% of its maximal activity even after 20 h of exposure. Following incubation at 70°C, the enzyme exhibited a half-life of more than 4 h, while XynDZ5 rapidly lost its catalytic activity when exposed to temperatures as high as 75°C (Figure 4). The determined optimum temperature around 65–70°C, combined with its thermal stability properties, classifies XynDZ5 as a natural thermophilic enzyme, suitable for a number of high-temperature applications (Collins et al., 2005; Basit et al., 2018).

The effect of a variety of metal ions, detergents, solvents and reducing agents on the xylanolytic efficiency of XynDZ5 was also determined. When NH_4_^+^, Li^+^, sodium citrate, β-mercaptoethanol, dithiothreitol (DTT) and ethanol were added to the reaction at a concentration of 10 mM, XynDZ5 activity was practically unaffected (relative activity > 95%), whereas the addition of Ni^2+^, Cu^2+^, Cd^2+^, Pb^2+^, Cr^3+^, Co^2+^, Hg^2+^, Zn^2+^ and sodium dodecyl sulfate (SDS) at the same concentration inhibited the activity of the enzyme almost completely (measured relative activity < 3%) (Table 1). At 10 mM, Mg^2+^, Ca^2+^, Al^3+^ and ethylenediaminetetraacetic acid (EDTA) had a slightly inhibitory effect on the catalytic efficiency of XynDZ5 (relative activity measured > 75%), while the addition of Ba^2+^ and Mn^2+^ at the same concentration, resulted in a large reduction of the enzyme’s activity at about 34 and 21% of its maximal level, respectively, (Table 1). When the same modulators were tested at a concentration of 1 mM, the majority of them [Mg^2+^, Ni^2+^, Ca^2+^, Ba^2+^, Mn^2+^, Al^3+^, Cu^2+^, Pb^2+^, Cr^3+^, EDTA, phenylmethylsulfonyl fluoride (PMSF)] did not significantly affect the catalytic efficiency of XynDZ5 (relative activity > 75%) (Table 1). On the other hand, Ag^+^ and Hg^2+^ inhibited XynDZ5 completely, while the addition of 1 mM Cd^2+^, Co^2+^, Zn^2+^ and SDS, resulted in a decrease in the relative activity of the enzyme to 15, 16, 22, and 18% of its maximal level, respectively, (Table 1). Finally, after measuring the activity of XynDZ5 in the presence of 0.1 mM of the tested modulators, enzyme activity was found to be inhibited by the presence of Ag^+^ and Hg^2+^ (relative activity < 3%), was mildly affected by the addition of Pb^2+^, Zn^2+^ and SDS (relative activity > 75%), while the addition of Cd^2+^ and Co^2+^ at the same concentration resulted in a reduction of the relative activity, which was measured at about 67% and 63%, respectively, (Table 1).

**TABLE 1:** Effect of various effectors of the xylanolytic activity of XynDZ5.

Effector	Relative activity(%)*		
	
	Effector concentration (mM)		
	
	10	1	0.1
NH_4_^+^	103.6 ± 1.9	NM	NM
Mg^2+^	78.1 ± 0.8	94.6 ± 1.3	NM
Ni^2+^	3.0 ± 0.4	83.0 ± 7.6	NM
Ca^2+^	81.5 ± 0.7	99.3 ± 1.3	NM
Li^+^	106.8 ± 8.5	NM	NM
Ba^2+^	33.8 ± 1.2	98.0 ± 9.5	NM
Mn^2+^	20.8 ± 1.4	88.5 ± 2.1	NM
Al^3+^	88.8 ± 3.4	92.9 ± 1.7	NM
Cu^2+^	2.6 ± 0.3	97.0 ± 2.2	NM
Cd^2+^	1.9 ± 1.1	14.8 ± 1.3	67.4 ± 9.4
Pb^2+^	1.3 ± 0.5	70.2 ± 0.8	91.2 ± 3.0
Cr^3+^	0 ± 1.2	96.8 ± 1.6	NM
Co^2+^	1.6 ± 1.0	16.2 ± 1.0	62.9 ± 10.7
Ag^+^	NM	ND	3.6 ± 1.3
Hg^2+^	ND	ND	ND
Zn^2+^	1.3 ± 2.0	22.0 ± 0.6	86.4 ± 3.3
SDS	3.0 ± 0.3	18.0 ± 5.6	93.9 ± 9.9
Sodium citrate	101.6 ± 0.7	NM	NM
EDTA	78.9 ± 2.0	108.2 ± 4.0	NM
β-mercaptoethanol	98.5 ± 10.3	NM	NM
DTT	112.6 ± 1.0	NM	NM
Ethanol	101.2 ± 0.3	NM	NM
PMSF	NM	106.2 ± 0.9	NM

*Results are expressed as a percentage of the activity in the presence of a modulator, compared to the activity without any added compound in the assay mixture. Results represent the average of three measurements ± SD. NM = Not measured (when a modulator did not have an effect on the relative enzymatic activity at 10 mM or inhibited the relative activity at 0.1 mM, relative activities at additional concentrations were not measured). ND = Not detected.

**FIGURE 5:**
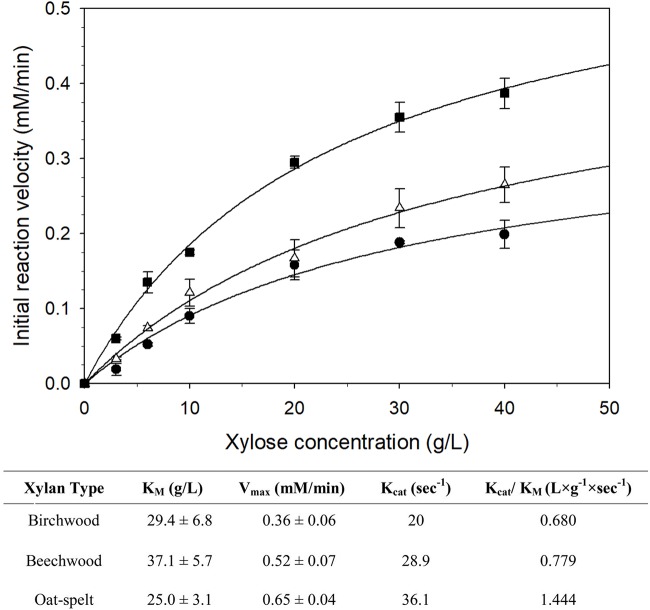
Michaelis-Menten plots for the determination of the K_M_ values of XynDZ5 for three xylan types. The initial reaction velocities (determined from the reducing sugars produced and expressed as equivalent xylose) were obtained in the standard assay mixture at pH 7 and 65°C with an enzyme concentration of 0.3 μM, birchwood xylan (∙), beechwood xylan (△), oat-spelt xylan (■). Data were fitted using the non-linear regression routines of SigmaPlot software. Values represent the mean of triplicate experiments and the error bars correspond to one standard deviation from the mean value. The table summarizes the kinetic constants determined from the above regression analysis.

When different xylans were tested as substrates for XynDZ5, oat-spelt xylan proved to be the most suitable one with K_M_ and k_cat_ values equal to 25 g/L and 36 s^–1^, respectively, (Figure 5). The K_M_ values for birchwood and beechwood xylan were relatively higher, but within the same range of 25–40 g/L. K_M_ values of similar magnitude have been reported for several fungal xylanases (Liao et al., 2014) but are significantly higher than the K_M_ values of most bacterial thermophilic xylanases (Cakmak and Ertunga, 2016).

**FIGURE 6:**
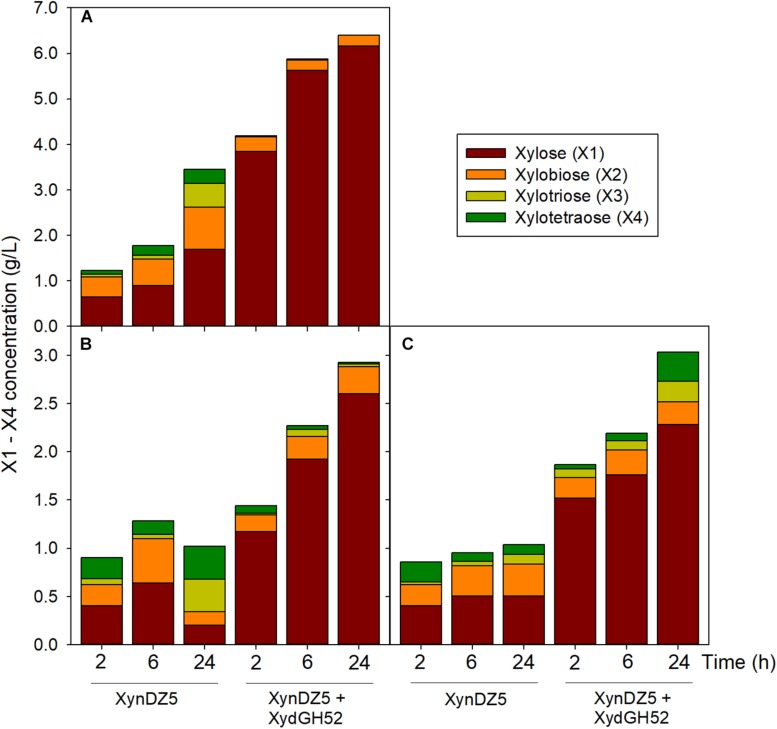
Reaction products following hydrolysis of various xylans with XynDZ5. XynDZ5 was incubated at 65°C and pH 7, either alone or with the addition of equimolar quantities of GH52 β-xylosidase of *Geobacillus* sp. SP24 (XydGH52). Samples were withdrawn at the indicated time intervals. Values represent the average of triplicate experiments. Standard deviations ranged between 2.7 and 18.6%. Beechwood xylan **(A)**, birchwood xylan **(B)**, oat-spelt xylan **(C)**.

Analysis of the reaction products of XynDZ5 revealed that, upon prolonged incubation of the enzyme with different xylans, the main xylooligosaccharide product was xylose (approximately 50%), followed by xylobiose (approximately 30%) and, in most of the cases, by xylotetraose (Figure 6). This pattern is quite different from the vast majority of endo-xylanases that produce mainly xylobiose and xylotriose upon action on various xylans (Collins et al., 2005; Chakdar et al., 2016). It also suggests that XynDZ5 has a significant exo-activity, removing xylose monomers from the reducing ends of the xylan backbone, and partially explains the high K_M_ and low k_cat_ values determined (Juturu and Wu, 2014).

Interestingly, a difference in the overall reaction kinetics was observed among the three types of the tested xylans. While for birchwood and oat-spelt xylan, the total amount of xylooligosaccharides produced, practically leveled off after 6 h of incubation, the reaction on beechwood xylan continued to release additional xylose to xylotetramers products for up to 24 h. This result probably reflects either different specific interactions of XynDZ5 with the corresponding xylan structures, or the formation of different reaction products from each xylan that possess variable inhibition effects on XynDZ5. The combined action of XynDZ5 with the GH52 β-xylosidase of *Geobacillus* sp. SP24 (XydGH52) (Galanopoulou et al., 2016), resulted in the alleviation of the inhibitory action of xylooligosacchrides on XynDZ5 and an up to 3-fold increase in the total X1 to X4 concentration, with xylose being the main product (80 – 95%) (Figure 6).

### Structural Modeling of XynDZ5

The amino acid sequence of XynDZ5 was analyzed with BlastP (Altschul et al., 1990) against the Non-Redundant (NR) and the UniProtKB/SwissProt protein sequence databases. When the XynDZ5 sequence was analyzed against the NR database, a 93% identity (96% query coverage) with a hypothetical 1,4-β-xylanase from *Thermoanaerobacterium thermosaccharolyticum* [NCBI Reference Sequence: WP_094397818.1] was detected. This result matches our original taxonomic analysis, which assigned the sequencing reads of the isolate to the genus *Thermoanaerobacterium.* BlastP analysis against the UniProtKB/SwissProt database, revealed that the closest characterized homolog of XynDZ5 is an endo-1,4-β-xylanase B from *Thermotoga neapolitana* [UniProtKB/Swiss-Prot: Q60041.1] (Velikodvorskaya et al., 1997) with sequence identity 26% (query coverage 70%). The second-closest sequence according to the BlastP results is another endo-1,4-β-xylanase from *Ruminiclostridium thermocellum ATCC 27405* [Uniprot accession no. A3DH97.1], with 25% sequence identity (query coverage 93%) (Wilson et al., 2013). Like XynDZ5, both *T. neapolitana* and *R. thermocellum* endo-xylanases originate from thermophilic bacteria and belong to the GH10 family.

In order to predict the three-dimensional (3D) structure of XynDZ5, computational studies were performed using two of the most widely used servers, the i-Tasser suite (Yang et al., 2015) and Phyre-2 (Kelley et al., 2015). Both servers predicted that XynDZ5 exhibits a (β/α)_8_ TIM barrel fold. More specifically, i-Tasser generated five models with a low confidence C-score ranging from −1.62 to −3.58, based on pairwise structure similarity clusters. Thus, Phyre-2 was employed instead, which utilizes the features of homology modeling, based on known protein structures and coupled with *ab initio* algorithms to model uncharacterized domains. Phyre-2 predicted 20 theoretical models with 100% confidence, with a sequence coverage ranging from 74 to 86% and a sequence similarity in the range 19–24%. The template structures used to generate the XynDZ5 model were superimposed and checked in terms of structural similarity. The model with the highest sequence coverage was selected; this was generated by employing the structure of an endo-β-1,4-xylanase belonging to GH10 family from *Cellvibrio japonicus* in complex with xylopentaose as a template (*Cj*Xyn10C-m with PDB ID 1us2, 86% coverage, residues 14-387 of XynDZ5) (Pell et al., 2004).

**FIGURE 7:**
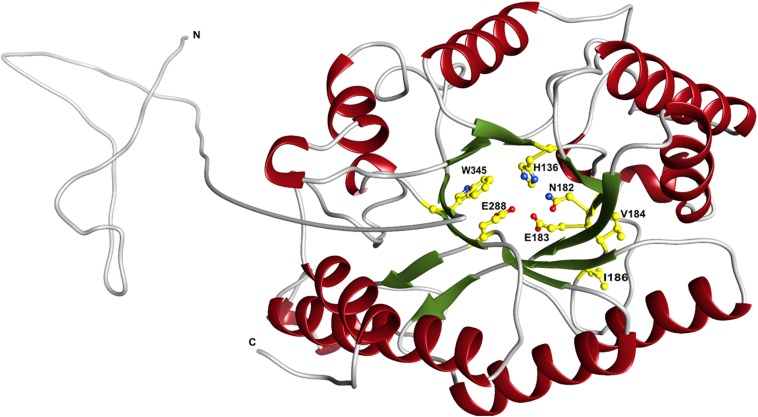
Schematic representation of the modeled XynDZ5 3D structure. The XynDZ5 3D structure was modeled using Phyre-2 presenting the secondary structural elements of the enzyme. The predicted catalytic dyad, E183 and E288, as well as the residues His136, N182, and W345 lying at the predicted active site are shown in ball-and-stick representation. *α*-helices and β-sheets are shown in red and green, respectively. In gray are the coiled areas and a non-modeled structure connected via a linker with the main enzyme. N and C are the N- and C- termini, respectively. The figure was prepared using UCSF Chimera (Pettersen et al., 2004).

The predicted XynDZ5 structure exhibits a typical (β/α)_8_ TIM barrel fold, consisting of eight α-helices and eight parallel β-strands that alternate along the peptide backbone (Figure 7). This type of structure is characteristic for several GH families, including both GH10 and GH11 xylanases, and is a feature found in glycosidase esterases as well (Henrissat et al., 1995).

**FIGURE 8:**
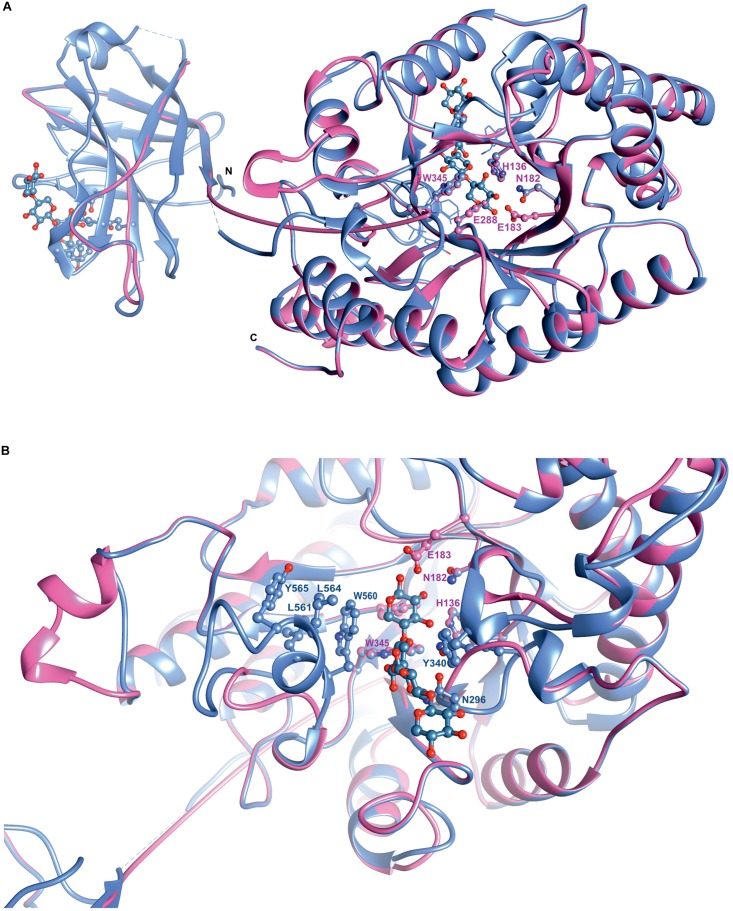
Superposition of the XynDZ5 modeled structure with its closest structural homolog. The modeled 3D structure of XynDZ5 shown in pink is superimposed on the 3D structure of its closest structural homolog *Cj*Xyn10C-m [PDB ID 1us2, Pell et al. (2004)] shown in cornflower blue using their corresponding secondary structural elements. In ball-and-stick representation are the conserved amino acids of the catalytic site and xylopentaose that binds to xylanase10C is shown in blue. N and C are the N- and C- termini, respectively, **(A)**. Top view showing the entrance of the catalytic site, highlighting the differences in the loop regions between XynDZ5 modeled structure and the structure from *C. japonicus*. Residues W560, L561, L564, and Y565 from *Cj*Xyn10C-m form a loop region closer to the catalytic site along with residues N296 and Y340 that stabilize the interactions with the ligand. These residues are not present in the model of XynDZ5 and the loops are in a different orientation, resulting in a more “open” site **(B)**. The figure was prepared using UCSF Chimera (Pettersen et al., 2004).

Superposition of the predicted model of XynDZ5 with the *Cj*Xyn10C-m template structure over secondary structural elements, showed that, although they follow a similar architecture, a total of about 40 additional amino acids, beyond those that form the TIM barrel structure, are modeled as a coil (Figure 8A). The catalytic site of the enzyme is composed of acidic and nucleophilic residues that belong to two of the β strands of the TIM barrel (Figure 7). The predicted catalytic dyad residues, E183 and E288, as well as the additional residues predicted to be part of the catalytic cleft, namely His136, N182, and W345 (numbering corresponds to the XynDZ5 sequence), are structurally conserved in XynDZ5 and *Cj*Xyn10C-m (Figure 8A). In the structure of *Cj*Xyn10C-m, however, there are additional amino acids that are implicated in hydrogen bonding and van der Waals interactions with the ligand upon binding. These residues adopt the same conformation in both the apo (PDB ID 1us3) and the ligand-bound structures (PDB ID 1us2), but they are missing from both the XynDZ5 sequence and modeled structure (Figure 8B). Furthermore, there are significant structural differences in certain loop regions between the *Cj*Xyn10C-m structure and the XynDZ5 model. More, specifically, the two loop regions comprising residues Gly295, Asn296 and Tyr340 belong to the substrate-binding site and stabilize the interactions formed, whereas in the modeled structure of XynDZ5, these loops point to different directions and away from the binding cleft, thus resulting in a more “open” site. In addition, the residues forming a hydrophobic cleft in the template structure (W560, L561, L564, and Y565; numbering corresponding to the *Cj*Xyn10C-m complex structure in the presence of xylopentaose) are not present in XynDZ5, suggesting that the residues dictating the orientation of the substrate upon binding might vary. Finally, *Cj*Xyn10C-m includes a glutamate/glycine substitution and a tyrosine insertion in the glycone region in its substrate binding cleft (Pell et al., 2004), which is not present in XynDZ5. Such polymorphisms in the substrate-binding cleft of GH10 enzymes have been suggested to affect substrate specificity (Pell et al., 2004). In accordance with this, the GH10 xylanase *Cj*Xyn10C-m displays activity against xylan in a fashion similar to XynDZ5, but it acts relatively poorly against xylooligosaccharides. In contrast with the vast majority of endo-xylanases that produce mainly xylobiose and xylotriose when hydrolyzing various xylans, XynDZ5 is differentiated as it exhibits significant exo-activity, which allows for the removal of xylose monomers from the reducing ends of the xylan backbone.

Regarding the pH profile of XynDZ5, previous studies performed with the *Bacillus halodurans* S7 xylanase (Mamo et al., 2009) revealed that residues V169, I170, D171 and their neighboring amino acids lying in the vicinity of the catalytic E168 (E183 in XynDZ5) are important for the hydrolysis of xylan at high pH. Out of these residues, both V169 and I170 (V184 and I186, respectively, in XynDZ5) are conserved in XynDZ5, thus providing a justification of the slightly alkalophilic nature of this new enzyme (Figure 7).

## Discussion

In the present work, we report the discovery and characterization of XynDZ5, a new thermostable xylanolytic enzyme with an attractive catalytic profile for various industrial biotransformations. *XynDZ5* was amplified from the genome of a pure isolate originating from a xylan-enriched culture, initiated from an Icelandic hot spring outflow material. XynDZ5 exhibits relatively low sequence similarity to previously characterized enzymes.

XynDZ5 belongs to the GH family 10 and acts optimally at 65–75°C and pH 7.5. This renders XynDZ5 an attractive biocatalyst for processes requiring thermostable and slightly alkalophilic xylanases, like the enzymatic pre-bleaching of paper pulp. Such enzymes are of great value as only few xylanases are reported to be active at both elevated temperatures and basic pH and usually originate from bacteria of the order Bacillales (Subramaniyan and Prema, 2000). Bacterial xylanases have been under the spotlight due to the fact that, until recently, the only xylanases that have been incorporated in the production pipeline of the leading xylanase manufacturing companies have been mainly of fungal origin and, thus, acidophilic. A few bacterial xylanases have been recently commercialized and these include Pulpzyme (manufacturer: Novozymes; application: paper industry), Propan BXC (manufacturer: Aumgene Biosciences; application: bakery) and Belfeed B1100 (manufacturer: Agrimex; application: feed additive).

XynDZ5-like xylanases are produced by several representatives of the genus *Thermoanaerobacterium*. The closest characterized homolog of XynDZ5 (26% sequence identity) is a GH10 xylanase originating from *Thermotoga neapolitana* (Velikodvorskaya et al., 1997). The *T. neapolitana* xylanase is an acidophilic enzyme (optimum pH 5.5), which remains stable at 70°C for up to 4 h of incubation. On the other hand, XynDZ5 is slightly alkalophilic and retains over 80% of its maximal activity even after 20 h of incubation at temperatures up to 65°C. The biochemical profile of XynDZ5 resembles that of the industrial GRE7 xylanase from *Bacillus* sp. GRE7 used for pulp bio-bleaching, whose optimal activity against oat-spelt xylan is 60–80°C and pH 7 (Kiddinamoorthy et al., 2008). Other reported bacterial enzymes presenting a profile similar to XynDZ5 are the thermostable and alkalistable xylanase from *Anoxybacillus flavithermus* TWXYL, with optimal activity at 65°C and pH 6-8 (Ellis and Magnuson, 2012), and the xylanase from *Streptomyces thermovulgaris* TISTR1948 with optimal activity at 65°C and pH 6.5 (Boonchuay et al., 2016).

An interesting feature of XynDZ5 is its significant exo-activity, which allows for the removal of xylose monomers from the reducing ends of the xylan backbone along with its typical endo*-*β*-1,4-*xylanase action. XynDZ5 exhibits a preference toward oat-spelt xylan compared to different xylans, with K_M_ and k_cat_ values equal to 25 g/L and 36 s^–1^, respectively. Although the K_M_ values for birchwood and beechwood xylans were relatively higher they remained within the range of 25 to 40 g/L, magnitude of value that is usually attributed to fungal xylanases (Liao et al., 2014), but are significantly higher than the K_M_ values reported for thermophilic xylanases of bacterial origin (Cakmak and Ertunga, 2016).

Structural modeling of XynDZ5 revealed that it folds into a typical (β/α)_8_ TIM barrel structure, which is common for GH10 xylanases. The predicted XynDZ5 fold was modeled based on the 3D structure of *Cj*Xyn10C, a GH10 xylanase from the phylogenetically distant bacterium *C. japonicus*. Superposition of the XynDZ5 predicted model with the *Cj*Xyn10C structure showed that the two enzymes share a similar architecture with conserved catalytic residues. Their main difference, however, is that in the *Cj*Xyn10C structure there are additional amino acids that are implicated in hydrogen bonds and van der Waals interactions, present both in the apo and the ligand-bound structure, which are missing from both the XynDZ5 sequence and 3D model. Furthermore, differences in loop regions result in a more “open” binding cleft in XynDZ5, probably contributing to altered enzyme specificity.

The influence of various effectors tested on the activity of XynDZ5 is similar with the reported for several other bacterial GH10 xylanases, such as the Xylanase A from *Clostridium acetobutylicum* ATCC 824 (Lee et al., 1993) and the Endoxylanase 2 from *Fibrobacter succinogenes* S85 (Matte and Forsberg, 1992). XynDZ5 activity is not affected by reducing agents such as β-mercaptoethanol and DTT, a fact that implies no interference with the catalytic action of the enzyme. According to the proposed XynDZ5 model structure, among the seven in total cysteines of the sequence, only two lie in the vicinity of the active site. Therefore, any potential changes in their oxidation state by reducing agents, at the concentrations examined, do not seem to affect the overall enzyme kinetics.

On the other hand, the inhibitory effect observed experimentally by heavy metals, such as Hg^2+^ and Ag^2+^, could be attributed to the formation of cysteine-conjugates with these metals that might affect neighboring residues inducing conformational changes that may lead to alterations or loss of the catalytic activity of the enzyme. (Pereira et al., 2017) (Supplementary Figure 2). This latter effect may additionally be attributed to the significant number of Trp residues of XynDZ5, where five out of twelve are located in the environment of the catalytic site, i.e. W345 that is located at the active site and four additional residues W104, W140, W178, W344 in the vicinity, according to the predicted 3D structure. Previous studies showed that the indole ring of Trp may undergo oxidation in the presence of mercury and silver, inducing conformational changes that could affect substrate binding (Pereira et al., 2017; Zhang et al., 2017).

Determination of the three-dimensional structure of XynDZ5 will provide more conclusive information on the biochemical interactions underlying these findings observations, and a better understanding of the catalytic mechanism of the enzyme.

Overall, XynDZ5 is a new candidate biocatalyst for biotechnological applications as it features characteristics favorable for various industrial setups that require xylanolytic action at high temperatures, neutral to basic pH and resistance to a variety of denaturing agents.

## Materials and Methods

### Reagents and Chemicals

All chemical reagents were purchased from Sigma-Aldrich unless stated otherwise. All molecular biology related products (restriction enzymes, protein markers, etc.) were from New England Biolabs.

### Environmental Sampling, Bioinformatics Analysis and Classification

The environmental sampling, enrichment process, DNA isolation and sequencing, as well as the bioinformatics analysis, have been described previously (Zarafeta et al., 2016a). Briefly, an environmental sample was retrieved from the outflow of a hot spring in Grensdalur, Iceland (64°01′53.4″N, 21°11′50.4″W). The temperature of the water at the sampling site was approximately 40°C and the pH ∼7. The sample was enriched anaerobically at 55°C, pH 7 with 0.01% (w/v) yeast extract and 0.5% (w/v) xylan as a carbon source. Following several sequential dilutions in xylan-containing medium, only rod-shaped microorganisms were visible under the microscope. The genomic DNA of an overnight culture of a pure isolate was retrieved and subjected to deep sequencing analysis. The raw sequencing reads were uploaded to our customized data analysis platform ANASTASIA (Koutsandreas et al., 2019). Assembly into contigs, *de novo* prediction of coding sequences within the contigs, and employment of three different types of integrated tools, each based on a different machine-learning model, were applied to identify 3,000 putative gene sequences, which were subsequently submitted to homology analysis.

Taxonomic classification of all assembled contigs was inferred with MEGAN (Huson et al., 2007). CAZome was identified by running all the obtained translated sequences against dbCAN database (release 6, last update Dec 2017) (Yin et al., 2012) with local HMMER v3.1b2^1^ (Finn et al., 2011). All queries with an E-value lower than 1 × 10^–18^ and signature domain coverage greater than 0.35 were accepted as true positives and were assigned to the CAZy family of the subject sequence.

The *in silico* determination of XynDZ5 domains was conducted with HMMER against Pfam-A database. The presence of putative transmembrane regions and signal peptides was investigated using TMHMM (Krogh et al., 2001) and SignalP (Petersen et al., 2011) accordingly.

### Plasmid Construction

The recombinant plasmid pASK75-*xynDZ5* was constructed by amplifying *xynDZ5* from the genomic DNA preparation retrieved from the xylan enrichment isolate by PCR. The following primers were used:

Forward: 5′-AAAAATCTAGAAGGAGGAAACGATGAG AGTAAATTTTATTTATAAAC-3′,

Reverse: 5′-AAAAACTCGAGTTA**GTGGTGGTGGTGGT GGTG**AATCGTGATTTCAATTGTTGTGC-3′

The forward primer contained an *Xba*I restriction site (underlined) and the reverse primer an *Xho*I restriction site (underlined) and a hexahistidine tag (bold). The amplification product was digested with *Xba*I and *Xho*I and inserted through a standard ligation reaction into a pASK75 vector (Skerra, 1994) digested with the same restriction enzymes. The correct sequence of the construct was verified by DNA sequencing.

### Protein Expression and Purification

*Escherichia coli* MC1061 cells carrying the plasmid pASK75-*xynDZ5* were grown in LB broth containing 100 μg/mL ampicillin at 37°C under constant shaking until the culture reached an OD_600_ of about 0.5. At that point, the overexpression of *xynDZ5* was induced by the addition of 0.2 μg/mL anhydrotetracycline followed by overnight incubation at 25°C. For XynDZ5 purification, the cells from a 500 mL culture grown in a 2 L shake flask were harvested, washed, re-suspended in 10 mL equilibration buffer NPI10, and lysed by sonication on ice. The cell extract was clarified by centrifugation at 10,000 × *g* for 15 min at 4°C and the supernatant was mixed with 0.5 mL of Ni-NTA agarose beads (Qiagen) and shaken mildly for 2 h at 4°C. The mixture was then loaded onto a 5 mL polypropylene column (Thermo Scientific), the flow-through was discarded, and the column was washed with two column volumes of NPI20 wash buffer. XynDZ5 was eluted using NPI200 elution buffer (200 mM imidazole). All buffers used for purification were prepared according to the manufacturer’s protocol (Qiagen). Imidazole was subsequently removed by gel filtration using a Sephadex G-25M PD10 column (GE Healthcare). Protein concentration was estimated by measuring absorption at 280 nm using the predicted extinction coefficient of the protein. The purified protein was visualized by SDS-PAGE analysis (Supplementary Figure 1).

### Enzyme Activity Assay

The xylanolytic activity of the enzyme was determined by quantifying the amount of reducing sugars released from beechwood xylan using the DNS method. The standard reaction consisted of 0.45 mL of a 10 g/L beechwood xylan suspension in 25 mM Tris–HCl buffer (pH 7.5), to which 0.05 mL of properly diluted enzyme sample were added. Reactions were carried out in plate on a thermal shaking platform at 70°C for 15 min unless stated otherwise. Upon incubation, reactions were terminated by the addition of 0.5 mL of DNS reagent and residual xylan solids were removed by centrifugation (5 min at 13,000 × g). Supernatants were transferred to new tubes and boiled for 5 min to allow color development. Aliquots of 200 μL were introduced into a micro-well plate and the absorbance at 540 nm was measured in a microplate reader against a blank sample, prepared the same way except that the enzyme was replaced by buffer. Linearity between reaction rate and working enzyme concentration was ensured by performing the assay using the appropriate enzyme dilutions so as not to exceed 10% of substrate conversion during the reaction. Xylanase activity was expressed in Units (U), defined as the amount of enzyme required to catalyze the production of 1 μmol of product per min under the above-described pH and temperature conditions.

### Biochemical Characterization

The optimal temperature for enzyme activity was determined by performing the standard enzyme activity assay at different temperatures ranging from 40 to 80°C. For the determination of the enzyme’s optimal pH, standard assay reactions were carried out at 50°C in 50 mM acetate, phosphate, Tris–HCl and glycine-NaOH buffers for pH values 4–6, 6–7.5, 7.5–9, and 9–10, respectively. Temperature stability of XynDZ5 was performed by incubating a properly diluted enzyme sample in 50 mM phosphate buffer pH 7 at various temperatures. Residual xylanase activity was determined at various time intervals. The effect of various modulators (metals, detergents, denaturants etc.) on XynDZ5 activity was determined by the addition of 0.1, 1.0, and 10 mM concentrations of the corresponding compounds under the standard assay conditions.

K_M_ and V_max_/k_cat_ values were determined for three different xylan substrates, namely beechwood, birchwood and oat-spelt xylan. Experiments were carried out under the standard assay conditions, using substrate concentrations ranging from 3 to 40 g/L. Data analysis and regression were performed using SigmaPlot.

Analysis of the reaction products resulting from the action of XynDZ5 on various xylans was performed using high-performance liquid chromatography. The experiments were carried out in a total volume of 1.6 mL at an initial xylan (birchwood, beechwood and oat-spelt) concentration of 25 g/L. The reactions took place at 65°C and pH 7 with the addition of either 0.2 μM XynDZ5 or equimolar quantities (0.2 μM) of XynDZ5 and the GH52 β-xylosidase of *Geobacillus* sp. SP24 (Stathopoulou et al., 2012). The latter enzyme has been previously isolated from the gDNA of the strain, overexpressed in *E. coli*, purified and partially characterized (Galanopoulou et al., 2016). Aliquots were removed from the reaction mixtures at specific time intervals, centrifuged, filtered and analyzed for their xylo-oligosaccharide (X1-X4) concentration in an Agilent 1220 HPLC system equipped with an RI 1260 Infinity detector. An APS-2 Hypersil column was employed (250 × 4.6 mm, Thermo Fisher Scientific), eluted at isocratic conditions with ACN/H_2_O, 70/30 (v/v).

### Modeling Studies

Modeling of the 3D structure of XynDZ5 (residues 1 to 430) was performed with i-Tasser (Yang et al., 2015) and Phyre-2 (Kelley et al., 2015). i-Tasser predicted models with low confidence score, C-score, ranging from −1.62 to −3.58 (default C-score range −5 to 2, for low to high confidence models), whereas the computed models by Phyre-2 had 100% confidence and sequence coverage up to 86%. The top 20 models from Phyre-2 were selected for further studies and emphasis was given to the one with the highest sequence coverage. Superposition using the secondary structural elements and molecular visualization of the structure with the closest structural homolog was performed by UCSF-Chimera (Pettersen et al., 2004).

## Data Availability Statement

The nucleotide sequence of *xynDZ5* can be found in the GenBank database under the accession number MN480471.

## Author Contributions

DZ, AG, DH, and GS conceived the idea for the project and designed the study. AG performed the genome analysis. DZ, AG, ML, and SK performed the biochemical experiments and analysis, MC and EC performed the modeling studies. DZ, AG, MC, FK, EC, DH, and GS were involved in the data interpretation, figures and table generation for the manuscript. DZ and GS wrote the manuscript with contributions from AG, MC, EC, and DH. All authors read and approved the final version of the manuscript.

## Conflict of Interest

The authors declare that the research was conducted in the absence of any commercial or financial relationships that could be construed as a potential conflict of interest.

## Footnotes

1 http://hmmer.org/
