# Supplementary material for: XynDZ5: A New Thermostable GH10 Xylanase
Source: Front Microbiol. 2020 Apr 24;11:545. doi: 10.3389/fmicb.2020.00545 (PMC7193231; doi:10.3389/fmicb.2020.00545)
Supplement: Supplementary file 1 [file Data_Sheet_1.pdf]

Supplementary Material

XynDZ5: A new thermostable GH10 xylanase

Dimitra Zarafeta[1†], Anastasia P. Galanopoulou[1,2†], Maria Evangelia Leni[1], Stavroula I. Kaili[2], Magda S. Chegkazi[1‡], Evangelia D. Chrysina[1], Fragiskos N. Kolisis[3], Dimitris G. Hatzinikolaou[2*], Georgios Skretas[1]*

[1]Institute of Chemical Biology, National Hellenic Research Foundation, Athens, Greece

[2]Department of Biology, Enzyme and Microbial Biotechnology Unit, National and Kapodistrian University of Athens, Zografou Campus, Athens, Greece

[3]Laboratory of Biotechnology, School of Chemical Engineering, National Technical University of Athens, Athens, Greece

[‡]Present address: Randall Centre for Cell & Molecular Biophysics, Faculty of Life Sciences & Medicine, King's College London, London, UK

[†] These authors contributed equally to this work

* Correspondence: G. Skretas (gskretas@eie.gr), D. G. Hatzinikolaou (dhatzini@biol.uoa.gr)

Supplementary Figures

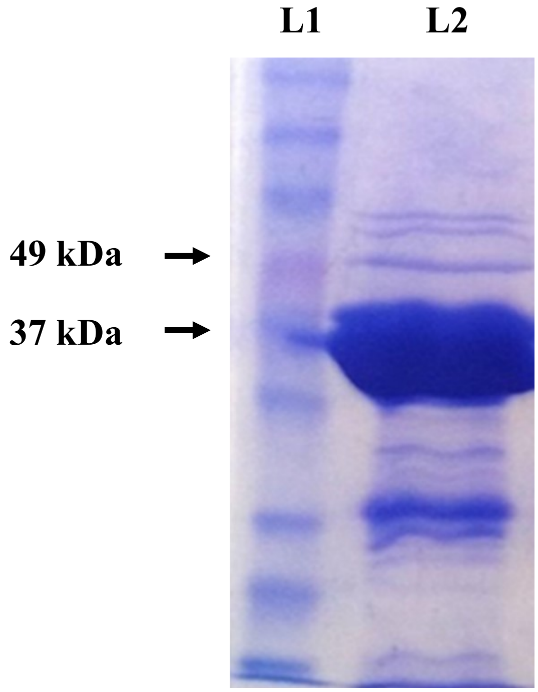

Supplementary Figure 1. SDS-PAGE analysis of purified XynDZ5. Protein bands were visualized by Coomassie staining. L1: molecular weight marker; L2: purified His-Tagged XynDZ5 (50.8 kDa).

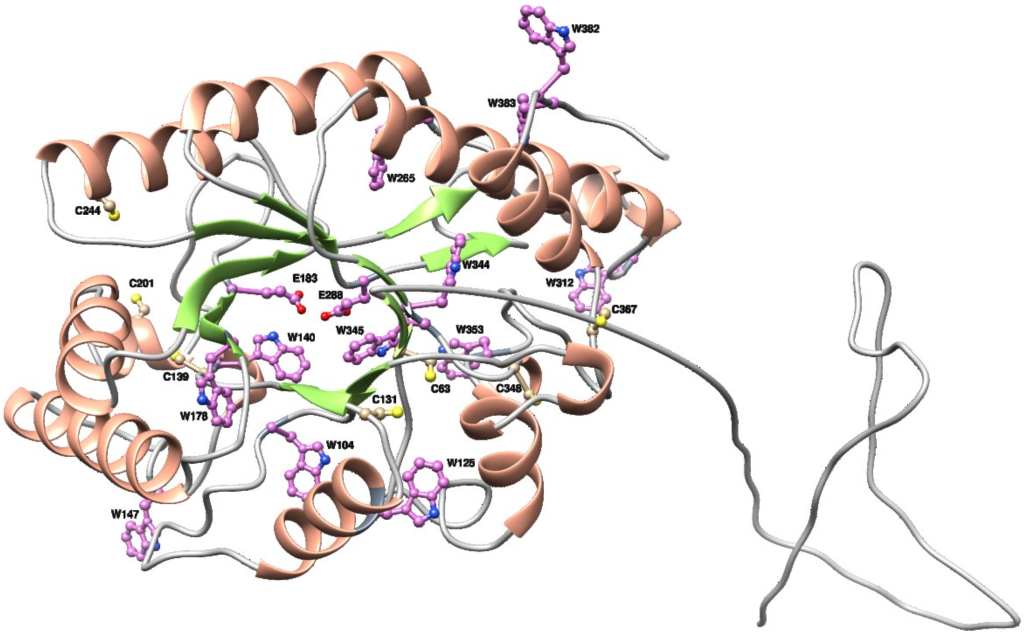

Supplementary Figure 2. Schematic representation of the modeled XynDZ5 3D structure. The predicted catalytic dyad E183 and E288, as well as the cysteine and tryptophan residues lying in the environment of catalytic site W104, W140, W178, W344, W345 are shown in ball-and-stick representation. *a*-Helices and *β*-sheets are shown in salmon and green, respectively. In grey the coiled areas and a non-modeled structure connected via a linker with the main enzyme are shown.
